# β→α Phase Transformation and Properties of Solid-State-Sintered SiC Ceramics with TaC Addition

**DOI:** 10.3390/ma16103787

**Published:** 2023-05-17

**Authors:** Shiyi Zheng, Buhao Zhang, Xuejian Liu, Zhongming Chen, Zhengren Huang, Jie Yin

**Affiliations:** 1 State Key Laboratory of High Performance Ceramics and Superfine Microstructures, Shanghai Institute of Ceramics, Chinese Academy of Sciences, Shanghai 200050, China; 2 School of Physical Science and Technology, ShanghaiTech University, Shanghai 201210, China; 3 Faculty of Engineering, University of Nottingham, Nottingham NG7 2RD, UK; 4 Ningbo Institute of Materials Technology and Engineering, Chinese Academy of Sciences, Ningbo 315201, China

**Keywords:** carbides, solid-state sintering, phase transformation, strength, toughness

## Abstract

Dense SiC-based composite ceramics were fabricated by means of the ex situ addition of TaC using solid-state spark plasma sintering (SPS). Commercially available β-SiC and TaC powders were chosen as raw materials. Electron backscattered diffraction (EBSD) analysis was conducted to investigate the grain boundary mapping of SiC-TaC composite ceramics. With the increase in TaC, the misorientation angles of the α-SiC phase shifted to a relatively small range. It was deduced that the ex situ pinning stress from TaC greatly suppressed the growth of α-SiC grains. The low β→α transformability of the specimen with the composition of SiC-20 vol.% TaC (ST-4) implied that a possible microstructure of newly nucleated α-SiC embedded within metastable β-SiC grains, which could have been responsible for the improvement in strength and fracture toughness. The as-sintered SiC-20 vol.% TaC (ST-4) composite ceramic had a relative density of 98.0%, a bending strength of 708.8 ± 28.7 MPa, a fracture toughness of 8.3 ± 0.8 MPa·m^1/2^, an elastic modulus of 384.9 ± 28.3 GPa and a Vickers hardness of 17.5 ± 0.4 GPa.

## 1. Introduction

Silicon carbide (SiC) ceramics have excellent properties, i.e., a high strength, high modulus, low thermal expansion, high thermal conductivity, and good corrosion resistance, enabling their widespread use in the nuclear, semiconductor and aerospace industries [1,2,3,4,5,6,7,8,9]. Densification of SiC ceramics is extremely difficult due to their strong covalent bonds, leading to a low self-diffusion coefficient (~10^−11^ cm^2^/s for C and ~10^−13^ cm^2^/s for Si) [10].

Classified by the state of the sintering additives during sintering, all these sintering approaches can be divided into liquid-phase sintering [11,12] and solid-state sintering [13]. The liquid-phase assisted sintering method is widely adopted to lower the sintering temperature of SiC ceramics, and the thermal and mechanical properties can be tailored by selecting and combining multiple sintering additives as well [4]. Liang et al. [14] obtained silicon carbide ceramics through liquid-phase sintering with Al_2_O_3_ and CeO_2_ as additives at 1840 °C, and the fracture toughness and flexural strength were 4.6 MPa·m^1/2^ and 437 MPa, respectively. Yi et al. [15] obtained a sample with a fracture toughness of 6.6 MPa·m^1/2^ through liquid-phase sintering with Al_2_O_3_-Y_2_O_3_ using the SPS method under a relatively low temperature of 1500 °C. Unfortunately, the considerable disadvantage of liquid-phase-sintered SiC ceramics is strength deterioration at certain temperatures, which inhibits their applications in harsh service environments and could have catastrophic consequences [16]. Additionally, the volatilization of the low-melting-point liquid phase during sintering would lead to massive amounts of waste and contaminate the lining materials of furnaces.

By contrast, diffusion-driven solid-state densification of SiC ceramics can be achieved using a limited range of additives. Al, B, C and B + C are all common sintering additives for solid -state sintering [17]. Additives can decrease grain boundary energy and eliminate oxides. As a high sintering temperature is necessary for solid-state-sintered SiC ceramics, the inevitable grain coarsening can lead to unideal mechanical properties [18,19]. Zhang et al. [20] obtained silicon carbide ceramics from gel casting and pressure-less sintering at 2200 °C. The grain size was in the range of 3–10 μm, while a few grains with a size in the 10–30 μm range were also observed, having the highest flexural strength of 531 MPa and a fracture toughness of 3.7 MPa·m^1/2^. Su et al. [21] reported the influence of in situ-synthesized AlN on the densification of SiC ceramics with a grain size of about 5–10 μm at 2170 °C. The flexural strength and fracture toughness of the sintered samples were 394 ± 48 MPa and 3.1 ± 0.2 MPa·m^1/2^, respectively. Chen et al. [22] obtained fully densified SiC/ZrB_2_ ceramics at 2300 °C with an average SiC grain size of 2.5–8 μm and a relatively low bending strength of 340 MPa. To date, a lot of work has been carried out with the aim of improving the strength and toughness of solid-state-sintered SiC ceramics. Li et al. [23] obtained a high fracture toughness of 7.0 MPa·m^1/2^ and a flexural strength of 458 MPa through the in situ formation of VB_2_ particles using a two-step sintering method at 1400 °C to form VB_2_ and 2150 °C for its densification. Huang et al. [24] reported that dense SiC/rGO ceramics were obtained via hot pressing at 2100 °C with submicron α-SiC as the starting powder, achieving a flexural strength of 625 MPa and fracture toughness of 7.9 MPa·m^1/2^. Sedlák et al. [25] obtained SiC/GPL composites via hot pressing at 2100 °C, achieving a fracture toughness of 4.4 MPa·m^1/2^, and plate-like graphene platelets were claimed to contribute to an increase in fracture toughness. The combination of SiC and TaC has shown great compatibility as well [26]. Hu et al. [27] obtained SiC-TaC composites with a flexural strength of 550 MPa at a sintering temperature of 1800 °C with 17.78 vol.% SiC using the SPS method. Attempts to increase the fracture toughness of ceramic via SiC-TaC were also made. Sharma et al. [28] obtained SiC-TaC composites with a fracture toughness of 3.85 MPa·m^1/2^ using the SPS method, and α-SiC and TaC were used as raw materials. The raw material of β-SiC tended to form more elongated grains than α-SiC, with mainly equiaxed grains, which resulted in better fracture toughness [29]. Liu et al. [30] obtained a fracture toughness of 6.8 MPa·m^1/2^ and a flexural strength of 703 MPa with 40 vol.% SiC at 1800 °C using the SPS method. The composite with TaC as the main phase had the disadvantages of high absolute density and an increased prevalence of harmful oxides forming inside TaC grains, leading to a decrease in mechanical properties.

However, few studies have reported using a strengthening and toughening strategy taking advantage of the β→α phase transformation of SiC. It is well known that the β→α phase transformation of SiC can lead to the in situ growth of elongated α-SiC grains [31]. It has been argued that the nuclei formation mechanism of α-SiC is influenced by the strain at the β/α interface, thereby preventing β→α phase transformation and the attendant formation of elongated grains [32,33]. Shao and Gu [34] reported core–rim structures of SiC-AlN undergoing α/β→α′/β′ transformation through sintering, which is beneficial to improve the fracture toughness.

In this study, the SPS method was used to fabricate a series of SiC-x vol.% TaC ceramics. Commercially available β-SiC powder was used as a raw material. The aim of the present investigation was to study the influence of TaC on the β→α phase transformation of SiC to describe the microstructure features of β-SiC/α-SiC-x vol.% TaC composite ceramics and to determine the strengthening and toughening mechanisms. Maintaining the phase transition ratio of β-SiC at the specific state in which α-SiC grains are elongated could increase fracture toughness while maintaining the high bending strength of unchanged small β-SiC grains. By using SPS, a rapid sintering method, and incorporating TaC, we successfully maintained the conversion fraction at an ideal state.

## 2. Materials and Methods

The raw materials used in the experiment were commercially available powders: β-SiC (D50 = 0.328 μm D90 = 0.679 μm, >99%, Enomaterial Ltd., Shanghai, China) and TaC (D50 = 1.20 μm D90 = 2.36 μm, >99%, Haoxinnano Ltd., Shanghai, China). The composition of the series of SiC-x vol.% TaC composite ceramics is listed in Table 1. To prepare the ceramics, the raw powders were weighed in calculated proportions; then, they were ball-milled continuously at 300 rpm for 6 h using SiC milling media and a ball-to-powder ratio of 1:1, using alcohol as a medium. The slurries were then dried at 60 °C for 12 h. The dried powder was then manually ground to pass through a 200-mesh sieve and put into a graphite mold with graphite paper as a liner, between upper and lower punches. After pre-compression under 20–30 MPa for 15 s, the mold covered with carbon felt was moved to a sintering chamber. The chamber was then sealed and evacuated. The heating process had two parts. First, the temperature was increased from room temperature (RT) to 1400 °C at a heating rate of 200 °C/min. Then, the heating rate was reduced to 100 °C/min till the temperature reached 2100 °C. The dwell time of each specimen was 10 min. During sintering, the temperature was measured and monitored with a thermocouple. Simultaneously, the pressure (40 MPa) was loaded during the soaking stage. The cooling rate was 100 °C/min until reaching 900 °C, and then 200 °C/min to RT. The final temperature was the same for all the compositions. The sintered specimens had the same dimensions of ~3 × Φ40 mm. A diamond emery cutter was used for removal of the graphite paper attached to the specimen for further characterization.

Theoretical densities of the series of SiC-x vol.% TaC ceramics were calculated according to the laws of the mixtures, β-SiC 3.2 g/cm^3^ and TaC 14.5 g/cm^3^. Open porosity and relative density were determined following Archimedes’ law. The phase analysis of the as-sintered ceramics was performed using X-ray diffraction (XRD, D8 Advance, Bruker, Karlsruhe, Germany) with Cu Kα radiation and an EIGER2 R detector. For all compositions, the XRD parameters were as follows: scanning range 10–80°, step size 0.02°, counting time 1°/min. Data for Rietveld refinements showed the strongest peak intensity of ~10,000 and sub-peak intensity of ~7000. X-ray diffraction was performed on a polished surface. The software Jade was used for Rietveld refinement. A field-emission scanning electron microscope (SEM, Magellan 400, FEI, Hillsboro, OR, USA) with energy-dispersive X-ray spectroscopy (EDS) and electron backscattered diffraction (EBSD) was utilized to observe the microstructure of polished and fractured surfaces.

Flexural strength and modulus were determined using a universal testing machine (Instron 5566, Instron, Norwood, MA, USA). The span was 16 mm and the crosshead rate was 0.5 mm∙min^−1^. Five specimens with dimensions of 1.5 mm × 4.0 mm × 18 mm were tested to obtain the averaged data. The face under tension was polished for bending tests. The Vickers hardness was measured using the indentation technique (TUKON-2100B, Instron Co, Norwood, MA, USA) using a load of 5 kg. The tested surface was polished. Fracture toughness (*K_IC_*) was evaluated using the single-edge notched beam (SENB) test at a crosshead rate of 0.05 mm∙min^−1^. The notch was made 2 mm perpendicular to the 1.5 mm × 18 mm surface using a diamond cutter. The average of 5 records was taken as the final result. The SENB method followed Equation (1) to estimate fracture toughness:

(1) $$K_{IC}=Y\frac{PL}{bW^{3/2}}\frac{3{\left(\frac{a}{W}\right)}^{1/2}}{2{\left(1-\frac{a}{W}\right)}^{3/2}}$$

*K_IC_*—fracture toughness (MPa·m^1/2^);
*P*—load (N);
*L*—span (mm);
*b*—width (mm);
*a*—notch depth (mm);
*W*—height (mm).

*Y* is a dimensionless factor correlated with *a*/*W* ratio; see Equation (2).


(2) $$Y=\frac{1.99-\frac{a}{W}\left(1-\frac{a}{W}\right)\left(2.15-3.93\left(\frac{a}{W}\right)+2.7{\left(\frac{a}{W}\right)}^{2}\right)}{1+2\left(\frac{a}{W}\right)}$$

## 3. Results and Discussion

**Figure 1:**
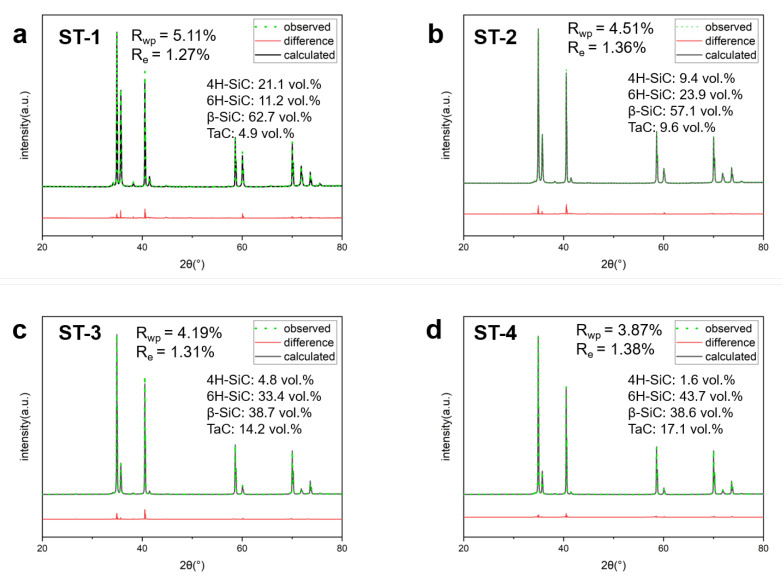
XRD Rietveld refinement results for (**a**) ST-1; (**b**) ST-2; (**c**) ST-3; (**d**) ST-4.

As mentioned earlier, the β→α SiC phase transformation will take place at temperatures above 1600 °C [35]. Obvious XRD characteristic peaks of β-SiC (3C-SiC, PDF#29-1129), α-SiC phases (6H-SiC, PDF#49-1428) and TaC (PDF# 035-0801) were detected in ST-1, 2, 3 and 4. As reported in the literature, the nucleation of α-SiC tends to occur inside the β-SiC grains, resulting in the strain-induced elongation of α-SiC grains during sintering [34]. To quantitatively investigate the β→α SiC phase transition in the as-sintered SiC-x vol.% TaC composite ceramics, in-depth XRD Rietveld refinement was conducted as shown in Figure 1. Noticeably, the conversion fraction from β-SiC to α-SiC was influenced by the added amount of TaC. 4H-SiC was considered to be an intermediate phase at a sintering temperature of 2100 °C. With less 4H-SiC, the sintering process was considered to be more complete. As TaC increased, the ratio of 4H-SiC content decreased from 21.1 vol.% to 1.6 vol.%, indicating the sintering process was promoted. The conversion fraction of β-SiC to α-SiC in ST-1 was 0.34, while it went up to 0.37, 0.45 and 0.53 for ST-2, ST-3 and ST-4, respectively. This implies that the ex situ addition of TaC might affect the subsequent growth of α-SiC grains due to the occurrence of extra pinning stress [27].

**Figure 2:**
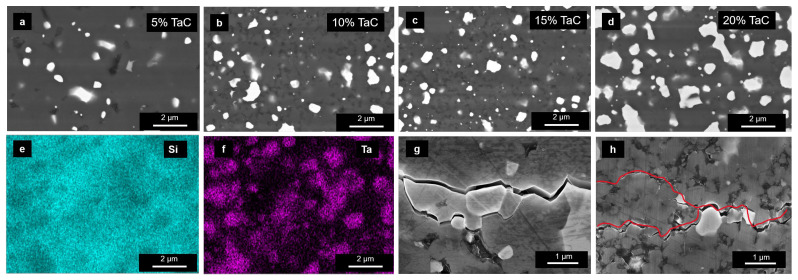
Backscattered SEM images of (**a**–**d**) SiC-x vol.% TaC ceramics with TaC volume ratios of 5%, 10%, 15% and 20%; (**e**) Si and (**f**) Ta distribution on the surface of ST-4; (**g**) intergranular fracturing and (**h**) fracture splitting after indentation test, the red line was drawn along the crack.

SEM images of SiC-x vol.% TaC ceramics are presented in Figure 2. SiC and TaC appear as gray and white, respectively, corresponding to their difference in atomic number contrast under the backscattered electron detector. EDS mapping was used to further confirm the element distribution of as-sintered SiC-20 vol.% TaC (ST-4). The gray region was identified as SiC, while the white region was identified as TaC, as the Ta element had an apparent distribution tendency. It is obvious that the fraction of the ‘white’ TaC phase grew with the increasing amount of additive among the as-sintered samples. The relative density of the series of SiC-TaC samples increased from 91.2% of ST-1 to 98.0% of ST-4, as shown in Table 1, which showed good consistency with the decreasing porosity (black pores). Fractures after the indentation test can be seen in Figure 2g,h. Intergranular fracturing and fracture splitting promoted fracture toughness.

**Figure 3:**
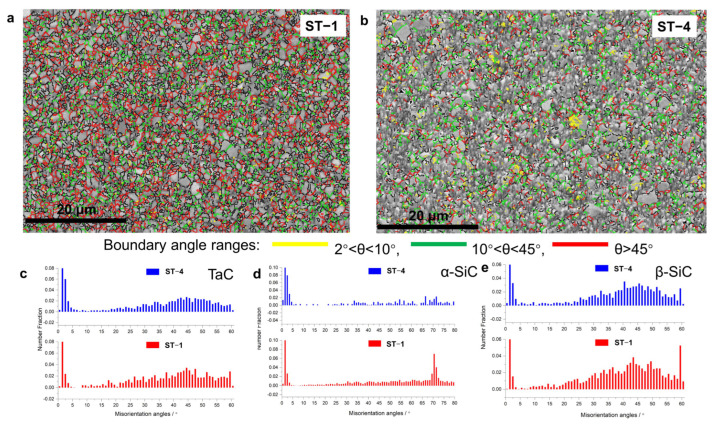
EBSD band contrast map with grain boundary of (**a**) ST1 and (**b**) ST4. The grain boundary misorientation distribution of (**c**) TaC, (**d**) αSiC and (**e**) βSiC.

The band contrast map with the grain boundary of ST-1 and ST-4 is shown in Figure 3. The misorientation angles of α-SiC, β-SiC and TaC were counted, and the results are shown in Figure 3c–e. Interestingly, as the TaC content increased, the high misorientation angles of α-SiC disappeared. It was deduced that the ex situ pinning stress from TaC greatly suppressed the growth of α-SiC grains and therefore led to its relatively low misorientation angle distribution. Newly formed α-SiC with finer grain sizes was therefore assumed to contribute to the mechanical properties. Fewer high-angle boundaries indicated low grain boundary energy, which could explain the improvements in the mechanical properties.

**Figure 4:**
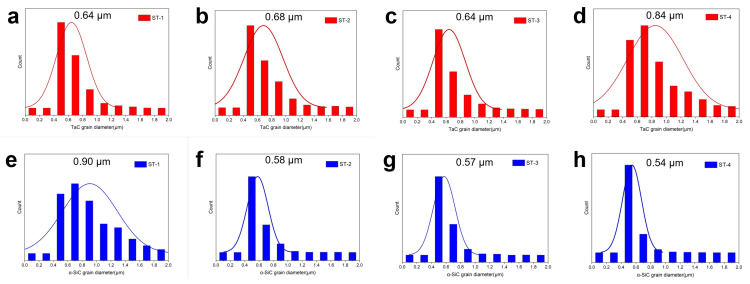
Grain diameter distribution of TaC in (**a**) ST-1, (**b**) ST-2, (**c**) ST-3, (**d**) ST-4 and α-SiC in (**e**) ST-1, (**f**) ST-2, (**g**) ST-3 (**h**) and ST-4 based on the EBSD test.

Grain diameter distribution of TaC and α-SiC were shown in Figure 4. Noticeably, a more significant grain growth of the TaC phase was observed from around 0.64 μm for ST-1 to around 0.84 μm for ST-4, while the grain diameter of α-SiC decreased from around 0.9 μm for ST-1 to around 0.54 μm for ST-4. The pinning effect can slow down grain boundary migration by exerting a pinning pressure that counteracts the driving force pushing the boundaries of the matrix [31]. An increased number of dispersed TaC particles can better maintain the grain boundary and hinder grain boundary migration more efficiently, which in turn would suppress the grain growth of α-SiC in ST-4.

The mechanical properties of as-sintered SiC-x vol.% TaC ceramics are shown in Table 1. The bending strength and fracture toughness increased significantly with the increasing addition of TaC: the strength increased from 455.5 ± 52.5 MPa (ST-1) to 708.8 ± 28.7 MPa (ST-4). According to the Hall–Petch theory (σ∝d^−1/2^), an increase in strength could be expected for the ST-4 sample due to the finer grain size distribution of α-SiC. The relative density of ST-4 was higher than that of ST-1, which would, on the other hand, increase the strength. Specifically, the fracture toughness increased remarkably, from 4.5 ± 0.8 MPa⋅m^1/2^ (ST-1) to 8.3 ± 0.8 MPa⋅m^1/2^ (ST-4). With a greater TaC content, the conversion ratio increased, causing the further formation of α-SiC and an improved fracture toughness. In addition, the modulus increased from 293.8 ± 12.5 GPa (ST-1) to 384.9 ± 28.3 GPa (ST-4); the maximum and minimum hardness values were 17.5 ± 0.4 GPa (ST-4) and 13.9 ± 0.1 GPa (ST-2), respectively.

## 4. Conclusions

In summary, the SiC-based ceramics were densified at 2100 °C using spark plasma sintering with a series of TaC (5, 10, 15, 20) vol.% additions. The conversion fraction from β-SiC to α-SiC was influenced by the amount of TaC added. With an increase in TaC added, the grain diameter of α-SiC decreased from 0.7 μm to 0.45 μm. The ex situ pinning stress of TaC greatly suppressed the growth of α-SiC grains and therefore led to its relatively low misorientation angle distribution. The β→α transformability of SiC-20 vol.% TaC ceramics was 20.9%. The relative density increased from 91.2% of the SiC-5 vol.% TaC sample to 98.0% of the SiC-20 vol.% TaC sample. The increase in the TaC content contributed to the improvement in the mechanical properties, achieving a bending strength of 708.8 ± 28.7 MPa and fracture toughness of 8.3 ± 0.8 MPa·m^1/2^, an elastic modulus of 384.9 ± 28.3 GPa and a Vickers hardness of 17.5 ± 0.4 GPa. In this study, incorporating TaC into compost improved the mechanical properties indirectly by (1) promoting the sintering process and (2) increasing the phase transition ratio of β-SiC.

## Figures and Tables

**Table 1:** Composition and mechanical properties of SiC-x vol.% TaC specimens.

Specimen	Composition	Apparent Density (g/cm^3^)	Relative Density (%)	Bending Strength (MPa)	Elastic Modulus (GPa)	Fracture Toughness (MPa·m^1/2^)	Vickers Hardness (GPa)
ST-1	95% β-SiC + 5%TaC	3.45	91.2	455.5 ± 52.5	293.8 ± 12.5	4.5 ± 0.8	15.6 ± 0.1
ST-2	90% β-SiC + 10%TaC	4.02	92.4	485.1 ± 82.7	296.0 ± 15.7	6.0 ± 1.0	13.9 ± 0.1
ST-3	85% β-SiC + 15%TaC	4.69	95.6	516.4 ± 47.0	315.8 ± 19.3	6.9 ± 0.5	14.0 ± 0.2
ST-4	80% β-SiC + 20%TaC	5.37	98.0	708.8 ± 28.7	384.9 ± 28.3	8.3 ± 0.8	17.5 ± 0.4

## Data Availability

Data will be made available on request.
